# Sequence Diversity within the Capsular Genes of *Streptococcus pneumoniae* Serogroup 6 and 19

**DOI:** 10.1371/journal.pone.0025018

**Published:** 2011-09-16

**Authors:** Karin Elberse, Sandra Witteveen, Han van der Heide, Ingrid van de Pol, Corrie Schot, Arie van der Ende, Guy Berbers, Leo Schouls

**Affiliations:** 1 Laboratory for Infectious Diseases and Perinatal Screening, National Institute for Public Health and the Environment (RIVM), Bilthoven, The Netherlands; 2 The Netherlands Reference Laboratory for Bacterial Meningitis and Department of Medical Microbiology, Academic Medical Center, Amsterdam, The Netherlands; Naval Research Laboratory, United States of America

## Abstract

The main virulence factor of *Streptococcus pneumoniae* is the capsule. The polysaccharides comprising this capsule are encoded by approximately 15 genes and differences in these genes result in different serotypes. The aim of this study was to investigate the sequence diversity of the capsular genes of serotypes 6A, 6B, 6C, 19A and 19F and to explore a possible effect of vaccination on variation and distribution of these serotypes in the Netherlands. The complete capsular gene locus was sequenced for 25 serogroup 6 and for 20 serogroup 19 isolates. If one or more genes varied in 10 or more base pairs from the reference sequence, it was designated as a capsular subtype. Allele-specific PCRs and specific gene sequencing of highly variable capsular genes were performed on 184 serogroup 6 and 195 serogroup 19 isolates to identify capsular subtypes. This revealed the presence of 6, 3 and a single capsular subtype within serotypes 6A, 6B and 6C, respectively. The serotype 19A and 19F isolates comprised 3 and 4 capsular subtypes, respectively. For serogroup 6, the genetic background, as determined by multi locus sequence typing (MLST) and multiple- locus variable number of tandem repeat analysis (MLVA), seemed to be closely related to the capsular subtypes, but this was less pronounced for serogroup 19 isolates. The data also suggest shifts in the occurrence of capsular subtypes within serotype 6A and 19A after introduction of the 7-valent pneumococcal vaccine. The shifts within these non-vaccine serotypes might indicate that these capsular subtypes are filling the niche of the vaccine serotypes. In conclusion, there is considerable DNA sequence variation of the capsular genes within pneumococcal serogroup 6 and 19. Such changes may result in altered polysaccharides or in strains that produce more capsular polysaccharides. Consequently, these altered capsules may be less sensitive for vaccine induced immunity.

## Introduction

*Streptococcus pneumoniae*, the pneumococcus, is responsible for infections such as meningitis, pneumonia and otitis media. The main virulence factor of the pneumococcus is its polysaccharide capsule. It protects the bacteria against phagocytosis and plays an important role in colonization of the upper airways [1]. Based on the reaction of the capsule polysaccharides with antisera, over 90 serotypes are recognized [2], [3], [4]. The sugars of the polysaccharides and the linkage between these sugars differ for each of the serotypes. The genes encoding the polysaccharides are located within the capsular gene locus and have a similar arrangement in most serotypes. The locus is positioned between the genes *dexB* and *aliA* [5], [6]. At the 5′ end, next to *dexB*, the genes for regulation and translocation are located. These genes, designated *wzg*, *wzh*, *wzd* and *wze*, are relatively conserved in all serotypes [7]. The genes involved in the synthesis of the polysaccharide and encoding glycosyltransferases, flippases and polymerases are located downstream of the regulatory genes. The *wzx* encodes flippase which is responsible for the transport of the sugars across the cytoplasmic membrane. *Wzy* codes for the polymerase-activity responsible for the synthesis of the polysaccharides in the so-called wzy-dependent pathway [8], [9]. Virtually all different polysaccharides are synthesized by this pathway except serotype 3 and 37 polysaccharides which are synthesized by the synthase-dependent pathway, using the synthase-encoding gene *tts* which is located elsewhere on the chromosome [6], [10].

For some serotypes the sequence of the capsular locus was already available [5], [6], [11], [12] but in 2006 the sequences of the capsular locus for the known 90 serotypes were published simultaneously [7]. Recently, new serotypes were recognized based on the DNA sequence of the genes in the capsular locus, for example serotype 6D [2]. Nowadays, serogroup 6 consists of serotype 6A, 6B, 6C and 6D. The difference between serotype 6A and 6B is claimed to be based on only a single nucleotide in *wciP* [13]. Two other polymorphisms in *wciP* have been found to be associated with serotype 6A or 6B, but there is uncertainty whether they are serotype specific [14], [15]. The polysaccharides from serotype 6A and 6B isolates differ in the way rhamnose is linked to ribitol. The capsular locus of serotype 6C is similar to serotype 6A, except for *wciN* which is altered and is 200 base pairs shorter in serotype 6C than in serotype 6A [4]. The glucose in serotype 6A polysaccharide is substituted by galactose in serotype 6C [16]. The capsular locus of serotype 6D is similar to that of serotype 6B but it contains the same altered *wciN* gene found in serotype 6C [2].

The capsular gene loci of serogroup 19 were among the first to be fully investigated [17]. The capsular genes of serotype 19F are quite similar to those of 19A and also serotype 19B and 19C have quite similar capsular genes. The differences between serotype 19A and 19F in the polysaccharides is based on the linkage between trisaccharides and *wzy* is thought to account for this difference in linkage [17], [18]. The serotypes 19B and 19C contain an additional side chain compared to serotypes 19A and 19F and have additional genes encoding these side chains [17].

In a study to assess the pneumococcal population in the pre-vaccination era in the Netherlands, capsular sequence typing (CST) revealed discrepancies between the phenotypic and genotypic serotyping within serogroup 6 [19]. CST is a molecular typing method to assess the serotype of a pneumococcal isolate and is based on a 506 base pairs sequence of the *wzh* gene. Serotype 6B and 19F are included in 7-valent pneumococcal conjugate vaccine (Pfizer Inc., Philadelphia, PA; PCV7), the vaccine that is currently used in the Netherlands. Epidemiological data show that serotype 19A is increasing in the USA [20] and also in the Netherlands after implementation of the vaccine in the national immunization program (unpublished data). This, in combination with the discrepancies found using CST between the phenotypic and genotypic serotyping within serogroup 6 prompted us to study these serotypes to gain insight in the sequence variation of the capsular loci within these serotypes and the role in vaccine induced immunity.

## Materials and Methods

### Isolates

Pneumococcal isolates from blood or cerebrospinal fluid in 2004, 2005, 2008 and 2009 were from patients with invasive pneumococcal disease and collected by the Netherlands Reference Laboratory for Bacterial Meningitis (NLRBM, Amsterdam, the Netherlands). Serotyping was performed at the NLRBM using the Quellung reaction as previously described [21], [22]. For molecular analyses bacteria were grown in 1 ml brain heart infusion broth with 0.5% yeast-extract overnight at 37°C and 5% CO_2_. Of each culture 500 µl was heated for 10 min at 95°C and these lysates were either used directly or stored at −20°C until use.

For comparison of the number of isolates within capsular subtypes in the pre- and post-vaccine era, only the strains isolated by 9 large medical microbiology laboratories, referred to as the sentinel laboratories, were used. This pneumococcal collection represents approximately 25% of all cases from Dutch patients with invasive pneumococcal disease in the Netherlands that occurred in the years 2004–2005 and 2008–2009.

### Sequencing of the complete capsular gene loci

The capsular loci of 25 isolates of serogroup 6 and 20 isolates of serogroup 19 were sequenced. Primer design was based on reference sequences [4], [7] and performed with Kodon 3.6 software (Applied Maths, Sint-Martens-Latem, Belgium). The PCR mixture of 20 µl contained Hotstartaq mix (Qiagen, Hilden, Germany), 10 µM of primer and 2 µl lysate diluted 1∶10 in sterile water. The PCR reaction was 15 min 96°C, 35 cycles of 30 sec 96°C, 1 min 50°C and 3 min 72°C followed by 10 min 72°C and for products <1 kb the PCR reaction was the same except 1 min 72°C instead of 3. PCR products were purified with ExosapIt (GE Healthcare Life Sciences, Chalfont St Giles, U.K.) according to manufacturer's protocol. One µl aliquots of the purified PCR products were used in the sequence reaction on an AB 3730 genetic analyzer using Big Dye Terminator technology (Life Technologies Corporation, Carlsbad, CA). The sequences of the complete capsular gene loci are deposited in Genbank (www.ncbi.nlm.nih.gov/Genbank) with the accession numbers JF911487–JF911531.

### Screening for serogroup 6 and 19 capsular subtypes

If one or more genes varied in 10 or more base pairs from the reference sequences published by Bentley et al. [7] and Park et al. [4], the isolate was designated as a capsular subtype. This threshold of 10 base pairs was arbitrarily chosen to allow for sufficient differences leading to amino acid substitutions. All serogroup 6 (n = 184), serotype 19A and 19F isolates (n = 195) in our collection were screened using allele-specific PCRs to identify the capsular subtypes. The allele-specific PCRs for serogroup 6 were created for the variable parts of *wzy* and *rmlC*, for serotype 19A for the variable parts of *wzg*, *rmlC*, *rmlB* and *rmlD* and for serotype 19F for the variable parts of *wzg*, *wze*, *wchA* and *rmlB*. For serogroup 6, the variations within the *wzg* and *rmlA* genes were assessed by sequencing.

### Genotyping

Multiple-locus variable number of tandem repeat analysis (MLVA) was performed as described in detail by Elberse and Nunes et al. [23]. Briefly, 8 variable number of tandem repeat loci were amplified in 2 multiplex PCRs in which one of the primers of each primer pair carried a distinct fluorescent label. The PCR products were mixed with a fluorescently labeled size standard (Life Technologies Corporation, Carlsbad, CA) and sized on an automated sequencer (AB 3730 genetic analyzer). Multi locus sequence typing (MLST) was performed as previously described [24], [25].

### Data Analysis

Alignment of the sequences was performed with Kodon 3.6. Data analysis and clustering were performed using Bionumerics version 6.5 (Applied Maths, Sint-Martens-Latem, Belgium). Tables with the MLVA profiles were imported from the Genemarker software into Bionumerics and the profiles were clustered using a categorical similarity coefficient and displayed in a minimum spanning tree. In a minimum spanning tree circles indicate the types. The size of the circle indicates the number of isolates with that particular MLVA type. The lines linking 2 types in the tree denote variants that differ from each other in a single VNTR locus (single locus variants). For the minimum spanning trees all entries from the database were used (n = 3592, database composition on December 20th, 2010) but only the serogroup 6 and 19 isolates were depicted. For assignment of MLVA complexes, the entire in-house MLVA database (available at www.mlva.net) was interrogated and the MLVA types with a single entry were excluded from the complex assignment. MLVA types belonged to a MLVA complex if their profiles differed in only a single VNTR locus. MLVA complexes were assigned as such only if they contained at least 3 MLVA types and a minimum of 9 entries. The discriminatory ability of the MLVA was measured using the Simpson's index of diversity (SID) and 95% confidence intervals were calculated as proposed before [26], [27].

## Results

### Diversity in the capsular genes of serogroup 6 and 19

**Figure 1:**
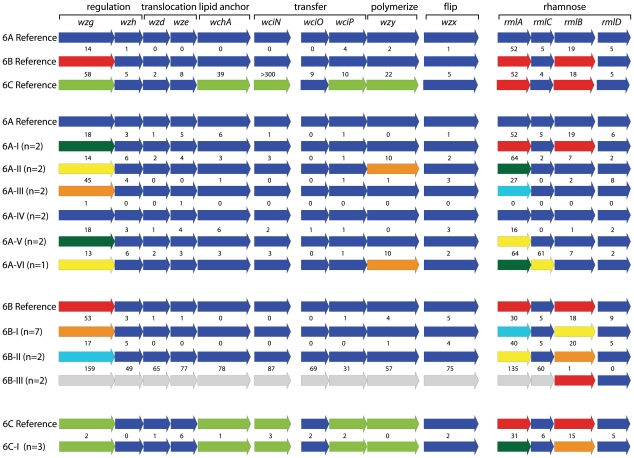
Schematic overview of the capsular gene loci of serogroup 6 isolates. The sequences were compared with the reference sequences [4], [7]. If 10 or more base pairs differed in one or more genes, a new allele number was assigned to the gene. The various alleles are indicated with colors that differ from the dark blue colors used for the reference genes.

The entire capsular locus of 11 serotype 6A isolates was sequenced and capsular subtypes were identified using the criterion of 10 or more nucleotide differences in one or more capsular genes from the reference sequence [4], [7]. Six distinct capsular 6A subtypes could be identified (Fig. 1, designated 6A I–VI). Major differences were found in *wzg* and in the rhamnose-genes, *rmlA*, *rmlC* and *rmlB*. Two isolates had sequences identical to the reference isolate with the exception of a single nucleotide and were called capsular subtype 6A-IV. An additional 64 serotype 6A isolates from our pneumococcal collection were screened by allele-specific PCRs and sequencing of the *wzg* and *rmlC* genes which led to another 6 new capsular subtypes (Table 1). The most frequently found capsular subtypes were 6A-I and 6A-II (23 and 22 isolates, respectively). The genes with the highest degree of sequence variation were *wzg*, *rmlA* and *rmlB*. The diversity index of serotype 6A capsular subtypes was 0.799 (0.747–0.850).

**Table 1:** Capsular subtypes within the serogroup 6.

			Sequencing (bp.)^1^		PCR^1^	
	n	Subtype	*wzg*	*rmlA*	*wzy*	*rmlC*
Serotype 6A (n = 75)	23	6A-I	4 (18)	3 (52)	1	1
	22	6A-II	5 (14)	2 (64)	2	1
	10	6A-III	1 (45)	1 (27)	1	1
	7	6A-IV	6 (01)	5 (00)	1	1
	4	6A-V	4 (18)	4 (16)	1	1
	1	6A-VI	5 (13)	2 (64)	2	2
	3	6A-VII^2^	1 (53)	7 (65)	1	1
	1	6A-VIII^2^	5 (14)	1 (27)	2	1
	1	6A-IX^2^	5 (14)	4 (16)	2	1
	1	6A-X^2^	9 (18)	3 (52)	1	1
	1	6A-XI^2^	5 (14)	1 (27)	2	1
	1	6A-XII^2^	8 (16)	1 (27)	1	1
Serotype 6B (n = 94)	87	6B-I	1 (53)	1 (30)	1	1
	3	6B-II	7 (17)	4 (40)	1	1
	3	6B-III	3 (159)	6 (135)	3	3
	1	6B-IV^2^	3 (159)	5 (52)	3	1
Serotype 6C (n = 15)	12	6C-I	2 (02)	2 (31)	2^3^	1
	3	6C-II^2^	2 (02)	3 (00)	2^3^	1

1 The various alleles are indicated with different numbers. Base pair difference compared to the reference sequences [4], [7] are indicated between brackets.

2 Subtype identified by screening only.

3 Differences between the serotype 6C and 6A *wzy* gene could not be detected with this PCR.

The serotype 6B isolates appeared to comprise a more homogeneous group (diversity index: 0.143 (0.046–0.240) than serotype 6A (Fig. 1 and Table 1). Ninety-three percent (87 of the 94 serotype 6B isolates) of serotype 6B isolates within our collection belonged to capsular subtype 6B-I. The main differences were found within the *wzg* gene and the *rml* genes. Remarkably, virtually all capsular genes in the 3 capsular subtype 6B-III isolates within the collection differed to a large extent from those in the reference sequence and from the capsular genes of other serotype 6B capsular subtypes. Screening of the serotype 6B isolates with allele-specific PCRs and specific gene sequencing resulted in a single additional new capsular subtype only. Based on the allele-specific PCRs, capsular subtype 6A-III and 6B-I were indistinguishable but according to the sequences of the entire capsular genes, there was a difference of 19 base pairs between the capsular subtypes.

The sequences of the complete capsular locus of 3 serotype 6C isolates were identical. However, the *rmlA* and *rmlB* genes in these isolates differed from those of the reference sequence described by Park et al. [4]. The screening of the additional 12 serotype 6C isolates within the collection yielded a single new capsular subtype. No serotype 6D isolates were found in our collection.

**Figure 2:**
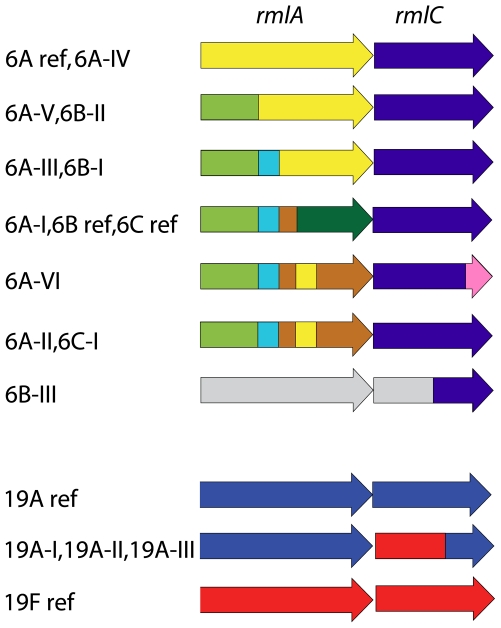
Mosaic structure indicating possible horizontal transfer within the *rmlA* and *rmlC* genes. Schematic overview of the *rmlA* and *rmlC* genes of serogroup 6 and 19. The various colors indicate similar sequences within the genes.

Parts of the *rmlA* and *rmlC* genes within serogroup 6 were found within all capsular subtypes, while other parts of the *rmlA* and *rmlC* genes were specific for a capsular subtype (Fig. 2). This may indicate that it is a possible recombination site within these genes. The sequence of *wzg* of serogroup 6 isolates can be divided into 3 classes. Capsular subtype 6B-III consists of a single *wzg* class. The remaining serogroup 6 capsular subtypes could be divided into 2 *wzg* classes, with 33 base pairs that were specific for each class. It seemed that a hotspot for mutations was present from base pair position 951–1116 within this gene. For example, the sequence of *wzg* of capsular subtype 6A-III differed from the reference sequence in 45 base pairs, and 78% (35) of the different base pairs were found in the mutational hotspot.

**Figure 3:**
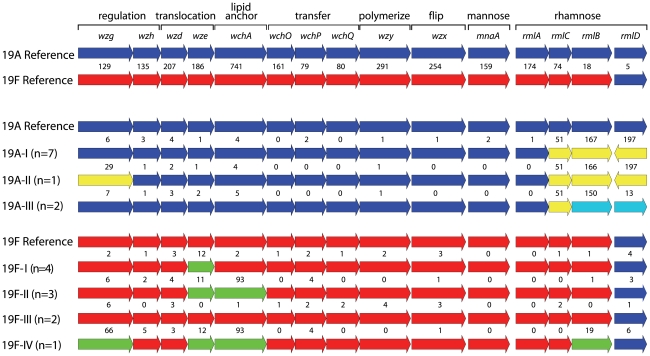
Schematic overview of the capsular gene loci of serogroup 19 isolates. The sequences were compared with the reference sequences [7]. If 10 or more base pairs differed in one or more genes, a new allele number was assigned to the gene. The various alleles are indicated with colors that differ from the dark blue (19A) and red (19F) colors used for the reference genes.

Within the sequenced capsular gene clusters of 10 serotype 19A isolates 3 capsular subtypes were identified (Fig. 3). Screening with allele-specific PCRs identified no new capsular subtypes within serotype 19A. Within our collection, 41, 38 and 35 isolates belonged to capsular subtypes 19A-I, 19A-II and 19A-III, respectively. The main differences between the capsular subtypes were found in *wzg*, *rmlC*, *rmlB* and *rmlD*. The first 363 base pairs of *rmlC* of the 19A capsular subtypes were identical to the sequence of the reference 19F isolate (Fig. 2). Furthermore, the *rmlD* gene of 19A-I and 19A-II appeared to be in the opposite orientation compared with the *rml* genes in all other sequenced capsular gene clusters.

Within the 10 sequenced serotype 19F isolates 4 capsular subtypes were identified. One capsular subtype, 19F-III, was rather similar to the reference sequence of Bentley et al. [7]. Screening by allele-specific PCR identified 44, 26, 10 and a single isolate belonging to capsular subtypes 19F-I, 19F-II, 19F-III and 19F-IV, respectively, but no new capsular subtypes within this serotype.

### Amino acid substitutions in the central region of the capsular locus of serogroup 6 capsular subtypes

**Figure 4:**
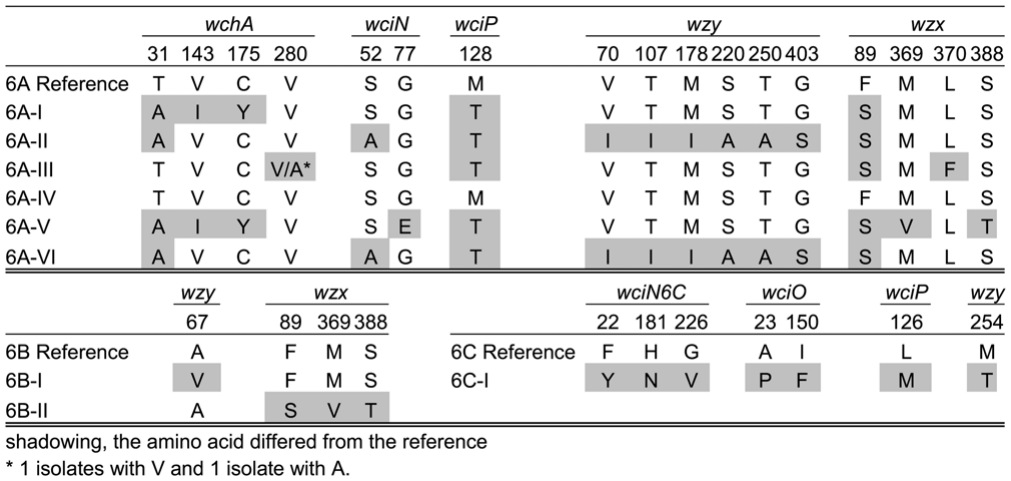
Amino acid substitutions in the alleles of the serogroup 6 capsular subtypes. The non-synonymous amino acid substitutions in the central region of the capsular locus of the serotype 6A, 6B and 6C subtypes are provided compared with the reference sequences [4], [7]. Subtype 6B-III was not included in this figure because of the extensive differences. Also, the capsular loci of serotype 6C differed significantly from serotype 6A and 6B isolates in *wciN* and therefore the *wciN* for serotype 6C was depicted as *wciN6C*.

The amino acid substitutions caused by changes in the genes involved in synthesis and export of the polysaccharides for the serogroup 6 capsular subtypes are depicted in Fig. 4. Capsular subtype 6B-III was not included in this figure, because of the extensive differences. Also, the capsular loci of serotype 6C differed significantly from serotype 6A and 6B isolates in the *wciN* gene and therefore the *wciN* gene for serotype 6C was named *wciN6C*. Within serotype 6A, capsular subtype 6A-II had the most amino acid substitutions and capsular subtype 6A-IV had no substitutions compared with the reference sequence. In *wchA* most non-synonymous substitutions were found in capsular subtype 6A-I and 6A-V. In the *wciN* and *wciP* genes, a single non-synonymous substitution in 6A capsular subtypes was identified. The *wzy* genes of capsular subtypes 6A-II and 6A-VI each had a 10 nucleotides difference compared with the reference sequence resulting in 6 amino acid changes.

Among the capsular subtypes of serotype 6B isolates only 4 non-synonymous substitutions were identified; one in *wzy* and 3 in *wzx*. Capsular subtype 6B-III was substantially different from the other capsular subtypes (Table 2). In all 6B-III genes involved in synthesis and export of the polysaccharides there were numerous non-synonymous substitutions. Most of these changes in amino acids were identified in *wzy* and *wchA* (29 amino acids), both caused by 34 nucleotide mutations. The lowest number of changes was found in *wciP*, and comprised 12 amino acid substitutions.

**Table 2:** Nucleotide substitutions and amino acid changes in subtype 6B-III compared with the 6B reference sequence.

	*wchA*	*wciN*	*wciO*	*wciP*	*wzy*	*wzx*
Synonymous substitutions	44	39	36	19	23	45
Non-synonymous substitutions	34	48	33	12	34	30
Number of amino acid substitutions	29	27	27	12	29	23

Three non-synonymous substitutions in *wciN* were identified in the serotype 6C isolates compared with the 6C reference sequence (Fig. 4). Also in *wciO*, *wciP* and *wzy*, 1 to 2 non-synonymous substitutions were identified. In *wzy*, all serotype 6C isolates showed a deletion of 3 residues at position 152 to 154 compared to serotype 6A and 6B sequences (data not shown).

In *wciP*, amino acids that distinguish serotype 6A from serotype 6B were found at position 192, 195 and 254 (Table 3). The serotype 6C isolates were identical in these positions to serotype 6A isolates. In a single serotype 6A isolate a non-synonymous substitution in position 192 was identified that was not consistent with the other serotype 6A isolates. This isolate belonged to capsular subtype 6A-IX, the only isolate identified within this subtype. The genetic background of this isolate is related to isolates within the MLVA complex comprising 6A-II and 6C-I isolates.

**Table 3:** Serotype specific important residues in the *wciP* gene of serogroup 6.

		Position amino acid		
serotype	n	192	195	254
6A	74	GCT (A)	AGT (S)	AGG (R)
	1	ACT (T)	AGT (S)	AGG (R)
6B	92	TCT (S)	AAT (N)	GGG (G)
6C	15	GCT (A)	AGT (S)	AGG (R)

### Amino acid substitutions in the central region of the capsular loci of serogroup 19 capsular subtypes

The serogroup 19 isolates differed only in a few amino acid residues from the reference sequences (data not shown). In capsular subtype 19A-I a single non-synonymous substitution in *wchA*, *wchP* and *wzy* was identified. In the capsular subtypes 19A-II and 19A-III there was a single non-synonymous substitution in *wchA* and in *wzy*, but the positions of the substitutions differed between both capsular subtypes. The *wze* gene of capsular subtype 19F-III was identical to the reference sequence but in capsular subtypes 19F-I, 19F-II and 19F-IV this gene was 2 amino acids shorter. Furthermore, capsular subtype 19F-I and 19F-II had 3 non-synonymous substitutions and 19F-IV had 4 non-synonymous substitutions in this gene compared with the reference sequence. The *wchA* gene of capsular subtype 19F-II and 19F-IV differed in 24 non-synonymous amino acid substitutions from the reference sequence. A few non-synonymous substitutions were identified in *wchO*, *wchP*, *wzy* and *wzx* in all of the 19F capsular subtypes.

### Genetic background compared with capsular subtypes

MLST and MLVA were performed on the isolates for which the capsular loci were completely sequenced. Both MLST and MLVA were performed because MLST is still considered the gold standard and MLVA can be easily performed on large scale. The correlation between MLST and capsular subtype was high for serogroup 6 subtypes, with some exceptions. The MLST profiles of the capsular subtype 6A-II isolates differed in a single locus (4 base pairs) from each other (Table 4). The MLVA profiles of these isolates were different in 3 loci, of which 2 loci could not be amplified, probably because of mutations in the primer sites and therefore the exact number of repeats is unknown. A single 6B-I isolate yielded a different MLVA type (MT 528 versus MT 524) while the ST (ST 176) was identical to other 6B-I isolates (n = 4). The 3 serotype 6C isolates had the same ST but different MTs.

**Table 4:** Sequence types and MLVA types of the capsular subtypes.

			MLST								MLVA (BOX loci)								
Serotype	Subtype	n	ST	aroe	gdh	gki	recP	spi	xpt	ddl	MT	1	2	3	4	6	11	12	13
6A	6A-I	2	681	2	5	9	1	6	19	14	578	4	3	12	5	2	1	1	5
	6A-II	1	327	1	5	7	12	10	1	14	697	5	3	99	99	2	1	1	6
	6A-II	1	3218	1	5	7	12	10	1	8	742	6	3	5	3	2	1	1	6
	6A-III	2	207	10	8	30	5	6	1	9	667	5	3	2	4	1	99	3	1
	6A-IV	2	65	2	7	4	10	10	1	27	66	1	2	7	3	3	1	4	3
	6A-V	2	1143	7	25	4	1	15	1	28	392	3	2	7	4	3	2	6	2
	6A-VI	1	327	1	5	7	12	10	1	14	691	5	3	12	99	2	1	1	6
6B	6B-I	2	138	7	5	8	5	10	6	14	245	2	3	2	2	3	2	7	5
	6B-I	4	176	7	13	8	6	10	6	14	524	4	2	2	3	2	2	6	6
	6B-I	1	176	7	13	8	6	10	6	14	528	4	2	2	3	2	2	99	6
	6B-II	1	497	7	25	4	2	48	20	28	406	3	2	12	4	0	1	7	2
	6B-II	1	new1	7	25	9	4	48	20	8	403	3	2	9	4	3	1	7	2
	6B-III	1	new2	7	6	1	8	6	1	14	413	3	3	1	3	2	2	1	5
	6B-III	1	90	5	6	1	2	6	3	4	775	7	2	6	3	2	2	1	2
6C	6C-I	1	1692	1	5	7	12	17	158	14	651	5	2	11	99	2	1	1	7
	6C-I	1	1692	1	5	7	12	17	158	14	870	5	3	99	3	2	1	1	6
	6C-I	1	1692	1	5	7	12	17	158	14	653	5	2	12	3	2	1	1	6
19A	19A-I	1	1521	10	43	4	1	6	4	8	69	1	2	7	4	2	2	5	5
	19A-I	1	193	8	10	2	16	1	26	1	846	99	2	9	2	1	1	1	6
	19A-I	1	416	1	13	14	4	17	51	14	68	1	2	7	4	2	2	5	3
	19A-I	1	423	1	5	4	12	5	3	8	71	1	2	7	4	2	2	6	5
	19A-I	1	199	8	13	14	4	17	4	14	535	4	2	6	1	1	99	2	1
	19A-I	1	1045	7	14	4	12	1	14	14	634	5	2	3	5	2	1	10	1
	19A-I	1	179	7	14	40	12	1	1	14	450	3	3	8	6	3	2	3	3
	19A-II	1	199	8	13	14	4	17	4	14	80	1	2	8	4	2	1	5	3
	19A-III	1	309	8	10	2	5	9	48	6	67	1	2	7	4	2	1	5	3
	19A-III	1	416	1	13	14	4	17	51	14	67	1	2	7	4	2	1	5	3
19F	19F-I	1	3008	1	5	14	4	17	51	14	296	2	3	10	99	2	2	9	1
	19F-I	1	1045	7	14	40	12	1	1	14	804	7	3	12	99	2	2	7	1
	19F-I	1	200	8	13	14	4	1	4	14	295	2	3	10	99	2	2	7	1
	19F-I	1	new3	5	5	62	5	9	3	19	434	3	3	5	3	2	1	12	2
	19F-II	1	172	7	13	8	6	25	6	8	68	1	2	7	4	2	2	5	3
	19F-II	1	876	8	13	14	4	6	4	14	480	3	4	7	6	0	1	5	3
	19F-II	1	new4	10	8	30	35	9	1	9	555	4	2	16	3	2	1	9	5
	19F-III	1	new5	2	27	2	4	9	6	31	162	2	2	1	2	2	1	6	1
	19F-III	1	new6	1	new	9	12	9	3	19	1005	3	2	6	3	2	1	5	4
	19F-IV	1	199	8	13	14	4	17	4	14	68	1	2	7	4	2	2	5	3

99, locus could not be amplified.

Both the MLST and the MLVA of the serogroup 19 isolates were highly diverse (Table 4). In the 10 serotype 19A isolates assessed by MLST, 8 different STs were found and 9 different MTs. There was no correlation between capsular subtypes and MLST or MLVA, except for MT67 in the capsular subtype 19A-III isolates. Also, the genetic background for serotype 19F isolates was very different, yielding 10 different STs and 9 different MTs within the 19F isolates. There were 4 new STs within the serotype 19F isolates that were yet unknown in the MLST.net database.

### MLVA for all isolates

**Figure 5:**
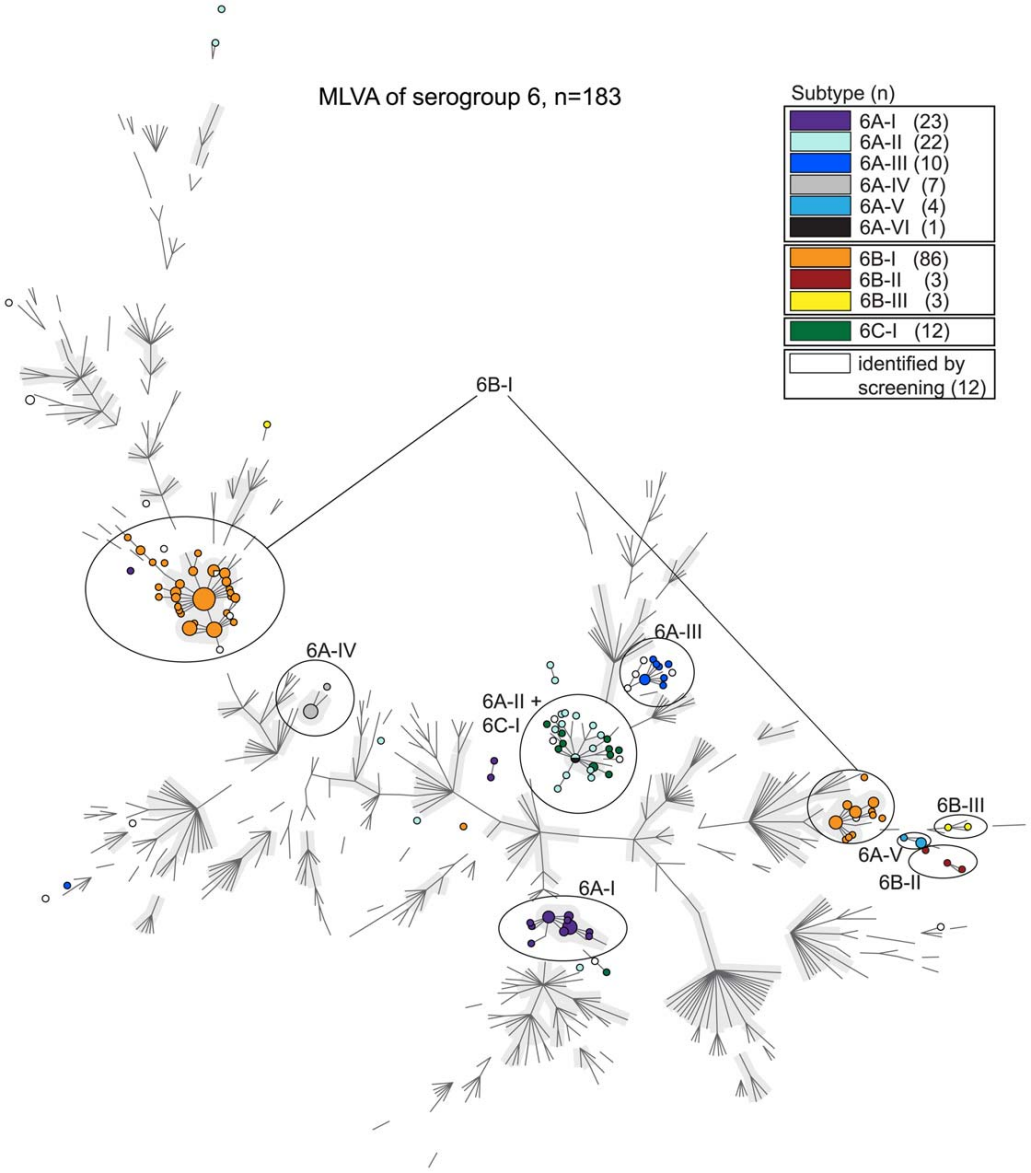
Relationship of the serogroup 6 capsular subtypes assessed by MLVA. Minimum spanning tree of the results obtained by MLVA for the 183 serogroup 6 isolates. Each colored circle indicates a genotype and the colors indicate the different capsular subtypes. Ellipses indicate capsular subtypes that have related MLVA profiles. The backbone of this minimum spanning tree is created using the entire MLVA database (n = 3592) (December 20, 2010), with only the branches made visible. The grey halos indicate MLVA complexes.

**Figure 6:**
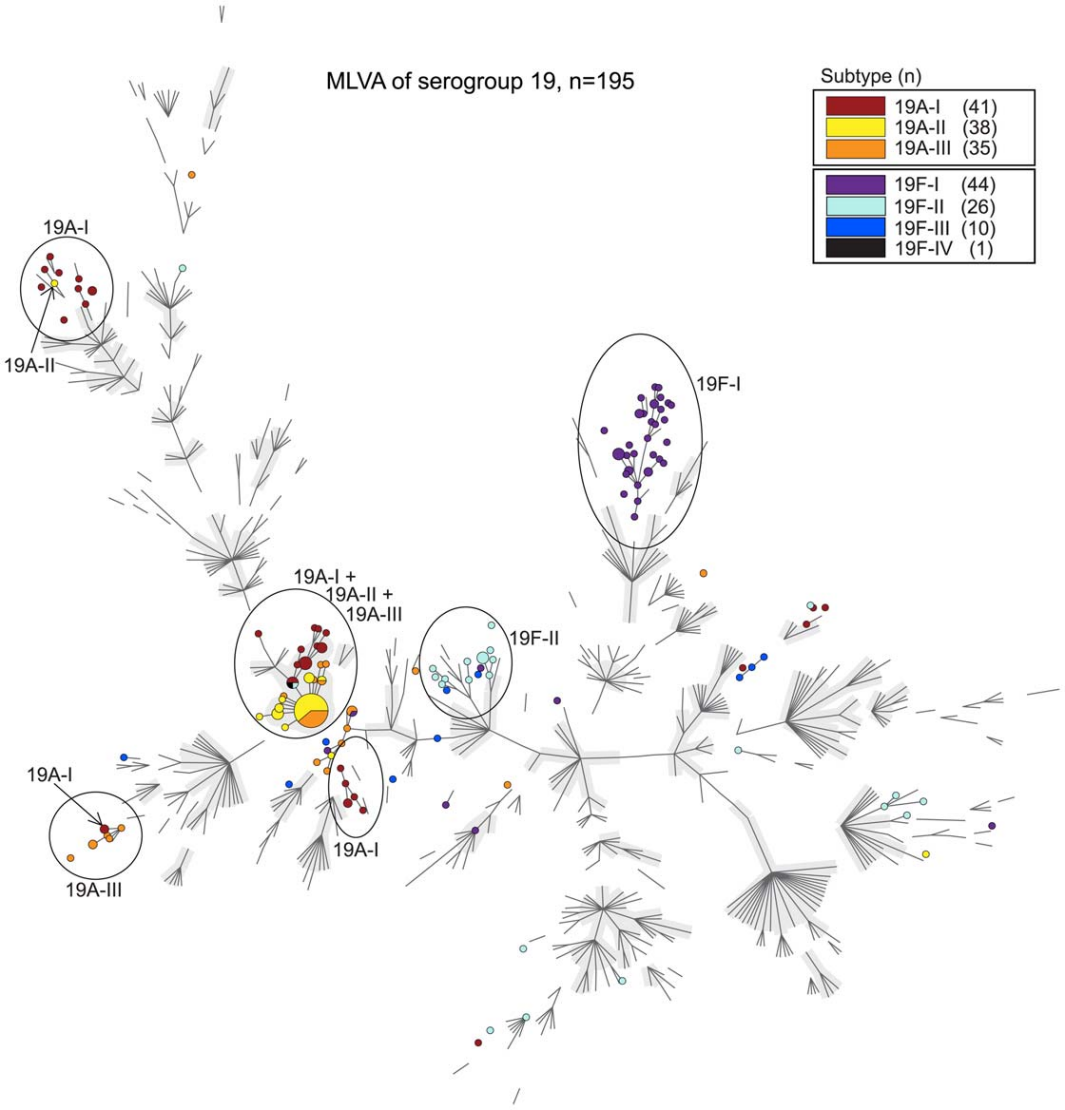
Relationship of the serogroup 19 capsular subtypes assessed by MLVA. Minimum spanning tree of the results obtained by MLVA for the 195 serotype 19A and 19F isolates. Each colored circle indicates a genotype and the colors indicate the different capsular subtypes. Ellipses indicate capsular subtypes that have related MLVA profiles. The backbone of this minimum spanning tree is created using the entire MLVA database (December 20, 2010), with only the branches made visible. The grey halos indicate MLVA complexes.

MLVA was performed for all serogroup 6 and 19 isolates (Fig. 5 and 6, respectively). The isolates within capsular subtype 6A-II and the serotype 6C-I isolates were closely related based on MLVA and MLST. Remarkably, capsular subtype 6B-I separated into 2 large MLVA complexes. The MLVA types within the 2 subtype 6B-I MLVA complexes differed from each other in 4 or more loci. The sequence of the capsular genes of the 2 MLVA complexes within the capsular subtype 6B-I isolates differed mutually in only 2 base pairs, but this did not lead to amino acid changes.

Despite the high degree of genotypic diversity as assessed by MLST and MLVA 52% of the serotype 19A isolates belonged to a single MLVA complex. The majority (84%) of the capsular subtype 19A-II isolates belonged to this complex. The capsular subtypes 19A-I and 19A-III were more diverse, because only 24% and 43% of these capsular subtypes belonged to that single complex, respectively. The MLVA of the serotype 19F isolates revealed a higher degree of diversity than the MLVA of 19A isolates. Of the 70 MLVA types found within the serotype 19F isolates, only 7 MLVA types were shared by 2 or more 19F isolates. The other 63 MLVA types included only a single serotype 19F isolate. Within the 19F-I capsular subtype 56% of the isolates were single locus variants of each other and clearly formed a group. However, this group did not meet the criteria to form a MLVA complex. Within the 19F-II capsular subtype, 46% of the isolates were genetically related based on MLVA. Other 19F isolates were scattered throughout the MLVA minimum spanning tree.

### Distribution of capsular subtypes before and after the introduction of the vaccine

In June 2006 PCV7 was introduced in the Netherlands. Shifts in capsular subtypes within serotypes after the introduction of the vaccine were observed (Table 5), although numbers were small. The number of cases caused by capsular subtype 6A-III had decreased from 7 cases in 2004–2005 to 1 case in 2008–2009. Also a decrease in capsular subtype 6C-I and an increase in capsular subtype 6C-II was observed. Remarkably, capsular subtype 19A-I accounted for 41% of the serotype 19A isolates in our collection in the pre-vaccination period and only for 24% in the post-vaccination period. In contrast, capsular subtype 19A-II increased within the post-vaccination period from 27% to 44%. Although these findings are noteworthy, the shifts are not statistically significant. Based on MLVA, there were 4 MLVA groups of serotype 19 isolates. The largest group contained isolates of capsular subtypes 19A-I, 19A-II and 19A-III. The capsular subtype 19A-I isolates, isolated after the introduction of the vaccine, were not present in this predominant serotype 19A MLVA complex, but were found throughout the MLVA minimum spanning tree and within the other 3 serotype 19A MLVA groups. The number of vaccine serotype 19F isolates significantly decreased after the introduction of the vaccine, in contrast with the isolates belonging to vaccine serotype 6B.

**Table 5:** Capsular subtypes of serogroup 6 and 19 in years pre-and post- vaccine introduction in the Netherlands.

		Total	2004–2005	2008–2009	
	Subtype	n	n (%)	n (%)	*p* value^1^
Serotype 6A	6A-I	18	9 (28)	9 (31)	1.000
	6A-II	18	11 (34)	7 (24)	0.414
	6A-III	8	7 (22)	1 (3)	0.055
	6A-IV	7	2 (6)	5 (17)	0.241
	6A-V	3	1 (3)	2 (7)	0.600
	6A-VI	1	1 (3)	0 (0)	1.000
	6A other	6	1 (3)	5 (17)	0.093
	Total 6A	61	32 (100)	29 (100)	0.697
serotype 6B	6B-I	73	36 (92)	37 (93)	1.000
	6B-II	2	1 (3)	1 (3)	1.000
	6B-III	3	2 (5)	1 (3)	0.615
	6B other	1	0 (0)	1 (3)	1.000
	Total 6B	79	39 (100)	40 (100)	1.000
Serotype 6C	6C-I	12	8 (100)	4 (57)	0.077
	6C other	3	0 (0)	3 (43)	0.077
	Total 6C	15	8 (100)	7 (100)	0.800
Serotype 19A	19A-I	31	17 (41)	14 (24)	0.079
	19A-II	37	11 (27)	26 (44)	0.095
	19A-III	32	13 (32)	19 (32)	1.000
	Total 19A	100	41 (100)	59 (100)	0.102
Serotype 19F	19F-I	39	26 (63)	13 (54)	0.601
	19F-II	18	11 (27)	7 (29)	1.000
	19F-III	7	3 (7)	4 (17)	0.409
	19F-IV	1	1 (2)	0 (0)	1.000
	Total 19F	65	41 (100)	24 (100)	*0.033*

1 Fisher exact test, 2 sided, italic if statistically significant (p<0.05).

## Discussion

In this study we investigated the sequence diversity of the capsular gene loci within the serotypes belonging to serogroup 6 (serotypes 6A, 6B, 6C) and serogroup 19 (serotypes 19A and 19F) of isolates from the Netherlands. We observed considerable variations among the capsular genes within serotypes which enabled us to assign capsular subtypes based on the sequence of the entire capsular locus. The largest number of capsular subtypes was found among serotype 6A, but in serotype 6B the highest degree of sequence variation between capsular subtypes was found. Capsular subtype 6B-III differed to a large extend from the 6B reference sequence and from the other 6B capsular subtypes. For both serogroup 6 and serogroup 19 most sequence diversity was found in *wzg* and the *rml* genes. Possible horizontal transfer of part of the genes was observed in *rmlA* and *rmlC* genes. Furthermore, in capsular subtype 19A-I and 19A-II, *rmlD* was oriented in the opposite direction compared with the rest of the capsular loci. Remarkably, we observed a decline in a serotype 6A capsular subtype and a switch between the capsular subtypes of 19A just 2 years after the introduction of PCV7 in the Dutch national immunization program.

The correlation between the genetic background as determined by MLVA and MLST and the capsular subtypes was high for serogroup 6. The isolates within a capsular subtype all had identical or closely related MLVA profiles. Only the isolates within capsular subtype 6B-I were separated into 2 MLVA complexes. Mavroidi et al. assigned *cps* profiles to the *wciP*, *wzy* and *wzx* genes of the serogroup 6 isolates based upon single nucleotide differences [13], [28]. MLST of these isolates showed considerable diversity and they found STs that included both serotype 6A and 6B isolates. The serotype 6A and 6B isolates in our study did not show overlap in MLST or MLVA. We verified whether the capsular subtype assignment would be different if we would assign the capsular subtypes in this study based on a difference of only a single base pair in the *wciP*, *wzy* and *wzx* genes, but the capsular subtype division of our collection remained unaltered. A possible explanation for the difference between Mavroidi's results and ours may be the large diversity in source of isolation and geographic origin of the isolates of Mavroidi et al. whereas we used only pneumococcal isolates isolated from patients with invasive disease within the Netherlands.

We identified 3 isolates of capsular subtype 6B-III in which the genes of the entire capsular locus differed by 943 base pairs (6%) from the serotype 6B reference sequence, resulting in 282 (5%) amino acid changes. In the genes involved in biosynthesis, 397 (5%) base pairs differed from the reference sequence, resulting in 147 (6%) amino acids changes. BLAST results revealed that this sequence was similar to an unpublished GenBank entry (AF246897). Also, this sequence is similar to a sequence designated as a class 2 sequence of serotype 6B by Mavroidi et al. and a class 2 sequence contains almost always an INDEL in *wciP* and differs in 5.4% from the reference sequence in *wciP*, *wzx* and *wzy* [13], [28]. As virtually all capsular genes differ from other serotype 6B capsular subtypes, we believe that capsular subtype 6B-III may represent a different serotype that expresses a capsular polysaccharide which cross-reacts with the 6B specific antiserum. After serological and biochemical characterization has confirmed the distinct nature of this variant it may well become ‘serotype 6E’.

Park et al. suggested that serotype 6C had evolved from a serotype 6A strain of which *wciN* was replaced by one of an unknown origin [4]. However, recently Bratcher et al. refined this theory and suggested that serotype 6C has evolved independently from serotype 6A. Serotype 6C would have evolved by the recombination of a large DNA fragment including both *wciN* and *wzy* from a nasopharyngeal gene pool that has not yet been defined [28]. However, our data show that the MLST and MLVA profiles of capsular subtype 6A-II and 6C are closely related and this would support the theory of Park et al. that serotype 6C is a descendent of serotype 6A.

According to literature, the difference between the capsule produced by serotype 6A and 6B strains is caused by a single nucleotide substitution in *wciP* at amino acid residue 195 [13], which was found in more than 100 serotype 6A and 6B isolates. Another position in *wciP*, amino acid residue 192, is thought to differentiate also between serotype 6A and 6B, but the data were inconsistent [13], [14]. Recently, position 254 in *wciP* was also marked as a serotype specific residue [15]. However, this was tested in 5 isolates only and again the outcome was inconsistent. Therefore, Sheppard et al. proposed that these isolates express a dual serotype due to an incomplete serotype switch. In our study, we assessed the sequence of *wciP* in 75 serotype 6A and 92 serotype 6B isolates and found that all 3 amino acid positions (192, 195 and 254) were serotype specific, except for a single 6A isolate at position 192. The positions 195 and 254 seemed to contain serotype 6A and 6B specific amino acids and therefore to distinguish serotype 6A from 6B, these 2 positions could be targeted for molecular identification. In this case, minimal sequence differences account for changes in polysaccharides and serotype. In our study, a threshold of 10 base pair difference in a gene is used to identify capsular subtypes. Smaller numbers of base pair differences are disregarded and thereby possibly relevant changes in the polysaccharides. However, clustering based on the sequences of the entire capsular loci of serogroup 6 and 19 isolates revealed the same subdivision compared to our threshold of 10 base pair difference in a gene.

There was a large diversity between the *wzg*, *rmlA* and *rmlB* genes between the isolates belonging to serogroup 6. Possible sites for horizontal transfer were observed in the *rmlA* and *rmlC* genes. It seems that within *rmlA*, there has been frequent transfer of part of the genes between the serogroup 6 capsular subtypes resulting in a mosaic structure. It seemed that the *rml* genes could be an important site for horizontal transfer, but we found no secondary mosaic site at the beginning of the capsular locus that could indicate the other recombination site required for transfer of the entire capsular gene locus. However, there is a mutational hotspot in *wzg*, but the capsular subtypes with the same mutations within *wzg* did not correspond with the capsular subtypes yielding the same *rmlA* and *rmlC* genes. Croucher et al. recognized that a serotype 6A capsular locus was inserted into a PMEN1 background. The sites for horizontal transfer were located in front of the capsular locus and within the *rml* genes [29]. Because we sequenced only the capsular genes and not the upstream region, such sites would not be detected in our study.

Our data showed that the *rmlD* genes of 79 serotype 19A isolates were oriented in the opposite direction compared to other sequenced *rml* genes, but also compared to the rest of the capsular genes. This gene arrangement was previously identified by Morona et al. and the promoter was found to be upstream of the *rmlD* gene [17]. Also in *Streptococcus mitis* and *Streptococcus oralis rmlD* is present in the opposite direction [30]. The finding that 69% (79 of 114) of the 19A isolates carried this *rmlD* gene suggested that this does not affect polysaccharide production or invasiveness.

The genetic background of serogroup 19 is known to be highly diverse [31], [32]. In our study, no clear relationship exists between the capsular subtypes of serogroup 19 and the genetic background. Five of the 10 STs that were found for serotype 19A isolates within our study were recognized previously in serotype 19F isolates according to the www.MLST.net database. This might indicate a horizontal transfer from serotype 19A capsular genes into a serotype 19F background. In a study performed by van Gils et al., nasopharyngeal colonization of serotype 19A was investigated using MLST. They found 2 serotype 19A isolates in vaccinated children with a sequence type previously associated with serotype 19F [33]. Selection of such variants in these studies might have occurred under pressure of the vaccine.

Noteworthy is the difference in distribution of some capsular subtypes before and after the introduction of the PCV7 vaccine. Although the vaccine does not contain serotype 6A, a decrease of the capsular subtype 6A-III occurred 2–3 years after vaccine introduction. The sequence of the capsular genes of subtype 6A-III was remarkably similar to the most prominent serotype 6B capsular subtype 6B-I which may suggest cross-protection. The capsular subtype 6A-III isolates used in this study were isolated from adults only suggesting that perhaps the already induced herd immunity could account for the decline in frequency of capsular subtype 6A-III, but we cannot exclude that normal fluctuations in the pneumococcal population could also account for the differences. Remarkable, but yet not statistically significant differences were seen in distribution of the serotype 19A capsular subtypes frequencies of the pre- and post-vaccine collection. The data suggest capsular subtype replacement from 19A-I to19A-II possibly indicating that capsular subtype 19A-II may be more successful in a human population where children are vaccinated against serotype 19F. The only difference between the capsular genes of 19A-I and 19A-II is found in the *wzg* gene. As *wzg* is involved in regulation of capsule production the 19A-II capsular subtype may have enhanced polysaccharide production. In a study of Weinberger et al. the serotypes with a higher degree of encapsulation were more resistant to neutrophil-mediated killing and more prevalent in carriage [34]. Therefore, higher production levels of the capsule would be advantageous in survival of the pneumococcus.

In conclusion, our study on the composition of the capsular genes of serogroup 6 and 19 revealed numerous substitutions within the serotypes. Changes within the capsular gene loci may result in altered polysaccharides or in increased production of the capsule making strains less sensitive for the vaccine induced immunity. We have isolated the polysaccharides of these serogroup 6 and 19 variants and are currently investigating the reactivity of vaccine induced and naturally induced antibodies with these polysaccharides to elucidate the effect of altered capsular genes on the structure of the capsule of the pneumococcus.
